# Exercise training-attenuated insulin resistance and liver injury in elderly pre-diabetic patients correlates with NLRP3 inflammasome

**DOI:** 10.3389/fimmu.2023.1082050

**Published:** 2023-02-01

**Authors:** Tan Zhang, Jingjing Tian, Jingcheng Fan, Xiangyun Liu, Ru Wang

**Affiliations:** ^1^ School of Exercise and Health, Shanghai University of Sport, Shanghai, China; ^2^ Shanghai Frontiers Science Research Base of Exercise and Metabolic Health, Shanghai University of Sport, Shanghai, China

**Keywords:** exercise training, NLRP3 inflammasome, pre-diabetes, insulin resistance, liver injury

## Abstract

**Background:**

Diabetes is one of the most common metabolic diseases and continues to be a leading cause of death worldwide. The NLRP3 inflammasome has been shown to exert detrimental effects on diabetic models. However, evidence linking NLRP3 inflammasome and pre-diabetes has been scarcely explored. Herein, we aimed to determine whether the NLRP3 inflammasome correlates with insulin resistance and liver pathology in a cohort of pre-diabetic subjects.

**Methods:**

50 pre-diabetic subjects were randomly assigned to a Pre-diabetes Control (DC, n=25) and a Pre-diabetes exercise (DEx, n=25) group. 25 Normal subjects (NC) were selected as controls. The DEx group performed a 6-month combined Yijingjing and resistance training intervention, while DC and NC group remained daily routines. Clinical metabolic parameters were determined with an automatic biochemistry analyzer; inflammatory cytokines were quantified by the ELISA assay; the protein expressions of NLRP3 inflammasome components in PBMCs were evaluated by Western Blot.

**Results:**

The insulin resistance, liver injury and NLRP3 inflammasome activity were higher in pre-diabetic individuals than in normal control group. However, 6-month exercise intervention counteracted this trend, significantly improved insulin sensitivity, reduced liver injury and inhibited the overactivation of NLRP3 inflammasome in pre-diabetic subjects. Moreover, positive correlations between insulin resistance, liver pathology and NLRP3 inflammasome were also found.

**Conclusions:**

Our study suggests that exercise training is an effective strategy to alleviate insulin resistance and liver injury in elderly pre-diabetic subjects which is probably associated with the inhibition of NLRP3 inflammasome activity.

## 1 Introduction

The prevalence of diabetes has increased worldwide, and it is predicted to affect almost 783.2 million people by 2045 (1). In addition, aging is one of the most serious public health concerns from this century, which further contributes to the occurrence and development of diabetes as the incidence of diabetes increases with aging (2). Thus, it is vital to elucidate the mechanisms underlying the pathogenesis of diabetes in elderly populations. Pre-diabetes is an intermediate stage between normal glycemia and diabetes and is highly prevalent, especially in older age groups and obese individuals. Although prediabetic individuals do not meet current thresholds for a diabetes diagnosis but they are at high risk of developing diabetes (3). Therefore, treatment of pre-diabetes is very promising for preventing the further progression of diabetes.

Considerable evidence has demonstrated that chronic low-grade inflammation caused by aberrant activation of the innate immune system plays an essential role in the pathogenesis of diabetes (4). Innate immune cells rely on pattern recognition receptors (PRRs) to recognize pathogen-associated molecular patterns (PAMPs) or damage-associated molecular patterns (DAMPs). The nucleotide-binding oligomerization domain-like receptor protein 3 (NLRP3) is an important intracellular PRRs. In 2002, Martinon (5) first proposed the concept of inflammasome. The NLRP3 inflammasome is the most widely studied inflammasome as far and it is comprised of the NLRP3, apoptosis speck-like protein containing a caspase recruitment domain (ASC) and precursor caspase-1 (pro-caspase-1). Under normal physiological conditions, the activity of NLRP3 inflammasome is extremely low to maintain a low inflammatory state. The activation of NLRP3 inflammaosme generally includes two steps: “priming” and “activation”. Priming: Once PAMPs or DAMPs are recognized by the PRRs, the nuclear factor kappa-B (NF-κB) translocation is triggered, which promotes the transcriptional expression of NLRP3, IL-1β and IL-18. Activation: The NLRP3 inflammasome can be induced by a variety of exogenous agonist including microbial components such as pathogenic nucleic acid (6), crystal and particles (7), and endogenous signals such as mitochondrial damage (8), cellular ion flux (9). In recent years, metabolic changes are also shown to negatively regulate NLRP3 (10). Upon stimulation, the NLRP3 first undergoes oligomerization and aggravates into the NLRP3 oligomers, then recruits ASC which subsequently recruits pro-caspase-1 and induces the self-cleavage of pro-caspase-1, lastly, the activated caspase-1 cleaves precursor interleukin-1β (pro-IL-1β) to from mature IL-1β which is released from cells to trigger inflammatory response (10). Therefore, aberrant activation of NLRP3 inflammasome leads to the maturation and secretion of pro-inflammatory cytokine IL-1β.

Over the past decade, it has been widely recognized that alterations in NLRP3 inflammasome activity is implicated in the pathogenesis of diabetes. In mice, ablation of NLRP3 prevented the obesity-induced inflammasome activation in fat depots and liver together with enhanced insulin-signaling (11). In addition, the NLRP3 inflammasome is tightly associated with liver inflammation and fibrosis. Global and to a lesser extent myeloid-specific NLRP3 inflammasome activation resulted in severe liver inflammation and fibrosis (12). On the contrary, the NLRP3 selective inhibitor MCC950 improved nonalcoholic fatty acid liver disease (NAFLD) pathology and fibrosis in obese diabetic mice (13). Similarly, NLRP3 gene deletion also ameliorated hepatic steatosis and inflammation in models of diet-induced obesity (14). Apart from these animal results, few studies have investigated the role of NLRP3 inflammasome in human models with diabetes. Previous studies found significantly increased mRNA and protein expression of NLRP3 and pro-inflammatory cytokines in monocyte-derived macrophages in subjects with diabetes when compared with healthy controls (15). Following studies confirmed these findings (16). However, although the importance of NLRP3 in diabetes has been established in animal and human models, the expression profiling of NLRP3 inflammasome in pre-diabetic subjects remains largely unknown.

The prevalence of diabetes is increasing rapidly and this is largely due to the alterations of lifestyle, in particular, excess energy intake and lack of physical activity are critical risk factors for diabetes. Therefore, to prevent pre-diabetes from developing into diabetes at an early stage, lifestyle intervention such as enhancing physical activity has been found to be very promising. Indeed, it has been well established that appropriate exercise is an effective strategy against diabetes but its underlying mechanisms remains unclear. Overwhelming studies have confirmed a strong association between physical inactivity and a moderate degree of chronic inflammation (17). Furthermore, recent studies showed that appropriate exercise was able to inhibit the overactivation of NLRP3 inflammasome in human models of T2DM (11), healthy young students (18), elderly subjects (19, 20), obese children (21). However, the evidence of beneficial effects of exercise intervention on inhibiting NLRP3 inflammasome activation in pre-diabetic human models is largely unknown (22). Moreover, in recent years, the traditional Chinese exercises including TaiChi, Yijinjing, Wuqinxi, Baduanjin, Liuzijue have been noted, whereas the research on Yijinjing is less. Similar to Taichi, Yijinjing is a traditional Chinese exercise based on traditional Chinese medicine theory, emphasizes the combination of symmetrical physical postures, meditative mind, and breathing techniques in a harmonious manner, and is consisted of linear movements and requires isolated joint movements. With simple patterns and directions, and moderate-intensity, safety, and efficacy, Yijinjing is easy to practice and is especially favored by the elderly populations. Previous studies showed that Yijinjing had the potential to improve cognitive function in patients with chronic schizophrenia (23) and post-stroke (24, 25), as well as ameliorate symptoms of people with knee osteoarthritis (26), ankylosing spondylitis (27). However, no randomized controlled trials (RCTs) are available to evaluate the effects of Yijinjing in patients with metabolic diseases such as pre-diabetes. In addition, according to the recommendation of American College of Sports Medicine (ACSM), combined aerobic and resistance training was the most effective in improving functional status of elderly subjects than aerobic or resistance training alone (28). Therefore, we aimed to explore the effect of combined Yijinjing and resistance training on pre-diabetic subjects and determine whether the NLRP3 inflammasome correlates with the pre-diabetic symptoms insulin resistance and liver injury.

## 2 Materials and methods

### 2.1 Study design and participants

The present study was a RCT trial. All participants provided written informed consent prior to the study. The sample size was determined based on similar literature (29). A total of 208 individuals were screened from Shanghai Yangpu district, of which 25 normal healthy subjects (NC) and 50 pre-diabetic patients were eligible. The pre-diabetic patients were randomly assigned to pre-diabetes control group (DC, n=25) and pre-diabetes exercise group (DEx, n=25) which is consistent with our previous study (30) and most of the other similar studies (29, 31).

The inclusion criteria were as follows: (1) men or women aged 50-70 years; (2) fasting blood glucose (FBG)<6.1 mmol/L and normal glucose tolerance for NC group; (3) 6.1≤FBG ≤ 7 mmol/L and overweight or obesity (waist circumference≥85 cm for men, waist circumference≥80 cm for women) for DC and DEx group. The exclusion criteria were as follows: (1) patients with cardiovascular, musculoskeletal or other kinds of diseases; (2) participants have no highly active lifestyle.

The daily physical activity and eating habits of all participants are strictly monitored by interviews every week. They were asked to maintain their routines without changing physical activity and eating habits. This study was approved by the Ethics Committee of Shanghai University of Sport (Clinical Trials ChiCTR2100054684).

### 2.2 Exercise training protocol

Individuals in the DEx group performed a 6-month combined Yijingjing and resistance training under the guidance of a professional Yijinjing instructor, five times/week for 6 months, while NC and DC group remained previous daily routines. The exercise intervention was conducted in Gongnong Park of Shanghai Yangpu district which was close to the subjects’ home from April to December, 2021. Yijinjing of Li Wei’s version was used in this study which was promoted by the General Administration of Sports of China with about 13 minutes. The type of resistance training used in this study was elastic bands exercise with about 8 minutes. All elastic bands were purchased from Li-Ning (China) Sports Goods Co., Ltd. The overload of elastic bands was 35 pounds (1500 mm x 1500 mm x 0.5 mm) for men and 25 pounds (1500 mm x 150 mm x 0.4 mm) for women. The exercise intensity was maintained at 60%-70% of the maximum heart rate with polar watches. Each exercise session consisted of a 5 min warm-up, Yijinjing training, elastic bands training and a 5 min cool-down period. The duration of exercise was increased from 47 minutes to 76 minutes per session. Briefly, in the first two month, two sets of Yijinjing training and two sets of elastic bands training were performed, with a total of 47 minutes per session. In the third and fourth month, three sets of Yijinjing training and three sets of elastic bands training were performed, with a total of 68 minutes per session. In the fifth and sixth month, three sets of Yijinjing training and four sets of elastic bands training were performed, with a total of 76 minutes per session.

### 2.3 Methods of measurement

#### 2.3.1 Anthropometry and physical examinations

All the measurements were performed at the baseline and 6 months after intervention at Shanghai University of Sport laboratory.

Background information regarding lifestyle, behavioral and motivational characteristics as well as medical history was collected. Height was determined using a wall-fixed measuring device, and weight was measured using a calibrated scale. Height and weight were used to calculate body mass index (BMI=weight(kg)/height(m)^2^). Blood pressure was measured with sphygmomanometer by professional physicians. Heart rate was determined by polar watches. Waist circumference and hip circumference were measured by a tape. Physicians examined the physical conditions of subjects and ensured that all the subjects met the inclusion criteria.

#### 2.3.2 OGTT test and metabolic parameters measurements

The oral glucose tolerance test (OGTT) was performed in according with the standard. Venous blood samples were taken after overnight fasting at 8:00-8:30 by physicians and at 2 h after the intake of glucose. FBG, fasting insulin (FINS), glycated hemoglobin (HbA_1c_), total cholesterol (TC), triglycerides (TG), high-density lipoprotein (HDL), low-density lipoprotein (LDL), alanine aminotransferase (ALT), aspartate aminotransferase (AST) and bilirubin, gamma-glutamyltransferase (GGT) and C-reactive protein (CRP) were measured from serum samples by an automatic biochemical analyzer. The homeostasis model assessment of insulin resistance (HOMA-IR) was calculated using the formula: [FBG x FINS/22.5].

#### 2.3.3 ELISA

The concentrations of pro-inflammatory cytokines tumor necrosis factor α (TNFα), interleukin-6 (IL-6) and IL-1β in serum were measured by ELISA assay using the human ELISA kits (Biolegend).

#### 2.3.4 PBMCs isolation

The activity of NLRP3 inflammasome was evaluated by detecting the protein expressions of NLRP3 inflammasome signaling cascade including NLRP3, IL-1β and caspase-1 in peripheral blood mononuclear cells (PBMCs). The PBMCs were isolated as described previously (32). In brief, venous blood samples were taken in standard fasting conditions at 8:00-8:30 by physicians, blood cells were separated from fresh blood and diluted in 1xPBS buffer, 4 ml diluted blood cells were added gently to a 15 ml centrifuge tube containing 2 ml lymphocyte separation medium ficoll and then centrifuged at 3000 rpm for 30 min at room temperature. The middle layer was transferred to a new centrifuge tube and washed with 1xPBS buffer. The remaining red blood cells were removed by adding 2-3 ml red blood cell lysis buffer and kept on ice for 5 min. The purified PBMCs were centrifuged at 1500 x g for 10 min at room temperature and cultured in RPMI-1640 medium.

#### 2.3.5 Western blot analysis

The activity of NLRP3 inflammaosme was detected by Western Blot analysis with whole-cell lysates and cell supernatants. Cells were washed with 1xPBS buffer and then lysed in RIPA lysis buffer with protease inhibitor (1 μg/ml) and incubated on ice for 20 min. Lysates were then centrifuged at 15,000 rpm for 15 min at 4°C. The concentration of protein supernatant was determined using the BCA protein kit. Total cell lysates were resolved by SDS-PAGE and analyzed for NLRP3, pro-caspase-1 and IL-1β protein levels. To examine secreted IL-1β and caspase-1, culture medium was collected, equal volume of methanol and 1/4 volume of chloroform was added and vortex. The mixtures then were centrifuged at 12000 rpm at 4°C for 10 min, the upper was discarded, the bottom was transferred to a new tube and equal volume of methanol was added and vortex. The mixtures were centrifuged at 12000 rpm at 4°C for 10 min, the supernatant was discarded and the pellet was dried for 5 min. 1xloading buffer was added to the pellet and then boiled at 95°C for 5 min. The cell culture medium lysates were resolved by SDS-PAGE and analyzed for cleaved caspase-1 and cleaved-IL-1β protein levels. 20-40 μg total lysate was separated by SDS-PAGE and transferred onto a PVDF membrane. Membranes were blocked with 5% non-fat milk and then incubated with primary and secondary antibodies respectively. The primary antibodies used in this study were NLRP3 antibody (abclonal A12694, 1:2000), caspase-1 antibody (Proteintech 22915-1-AP, 1:2000), IL-1β (abclonal A1112, 1:2000), and GAPDH antibody (Proteintech 60004-1-Ig, 1:50000). Western HRP Substrate (Millipore) was used for development of protein bands.

#### 2.3.6 Statistical analysis

The statistical analysis was performed with Graphpad Prism software V.8. The results are expressed as means ± SD. Paired t-test was used for intra-group comparisons before and after the intervention. Comparison among the three groups was performed by one-way ANOVA. The interactions between group and time were evaluated by two-way ANOVA. Correlation analysis was performed with Pearson’s test in the whole post-intervention samples. Statistical significance was set at *P*<0.05 or *P*<0.01.

## 3 Results

### 3.1 Participants and baseline characteristics

**Figure 1:**
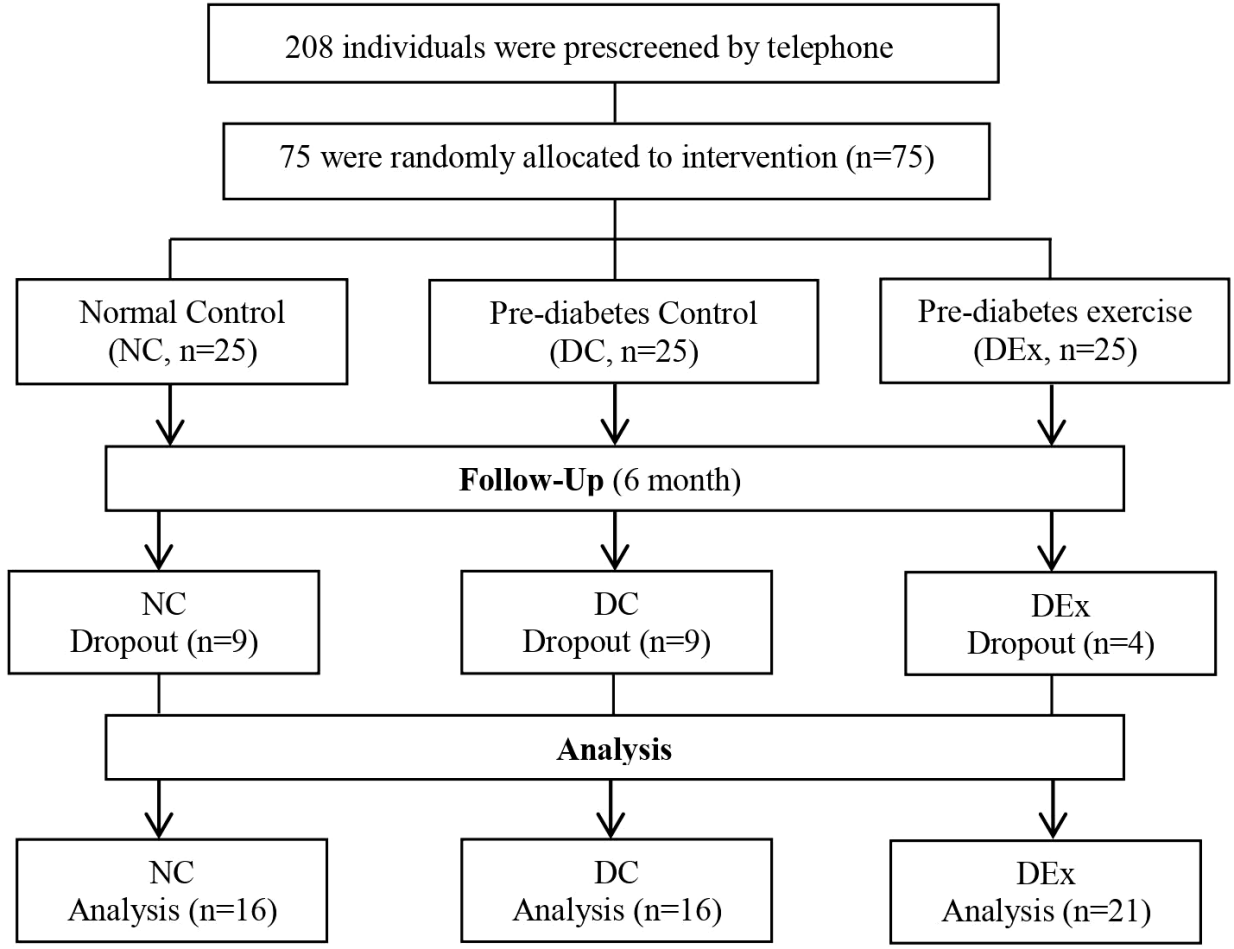
Study Profile of trial participants.

An overview of the study is given in **Figure 1**. We screened 208 individuals, of which 75 were eligible and assigned to NC (n=25), DC (n=25) and DEx (n=25) group. Of the 75 subjects, 53 subjects (71%) completed the interevention (n=16 in NC, n=16 in DC and n=21 in DEx group).The overall compliance was 64% for NC and DC gruop, 84% for DEx group, respectively.

The baseline characteristics of all groups were summarized in **Table 1**. All group had no significant differences in height, weight and BMI and waist-hip ratio, although individuals in the DC and DEx group tended to be fatter, the possible reason is that the objects in this study are pre-diabetic patients which are less severe than diabetes. Additionally, there was a significant difference in age between NC and DC subjects (*P*<0.05), however, no difference in age between NC and DEx group was detected. Moreover, the waist circumference (*P*<0.01) and hip circumference (*P*<0.05) were both significantly higher in DC sudjects as compared to NC group and the systolic blood pressure in DEx was also higher than in NC (*P*<0.05).

**Table 1:** Baseline characteristics of all groups.

Variables	NC (n=25)	DC (n=25)	DEx (n=25)
Sex (M/F, n)	8/17	8/17	7/18
Age (year)	59.44 ± 6.37	63.00 ± 4.72^*^	61.12 ± 6.57
Height (cm)	162.70 ± 10.01	165.18 ± 7.93	162.00 ± 7.34
Weight (kg)	63.71 ± 11.13	68.92 ± 10.96	64.66 ± 9.77
BMI (%)	23.89 ± 1.87	25.19 ± 3.01	24.90 ± 3.00
Waist circumference	85.74 ± 7.12	91.34 ± 7.09^**^	88.65 ± 7.37
Hip circumference	96.68 ± 7.62	100.95 ± 6.74^*^	100.18 ± 7.39
Waist-hip ratio	0.89 ± 0.07	0.91 ± 0.04	0.89 ± 0.05
Systolic blood pressure	131.64 ± 17.86	137.92 ± 17.77	142.17 ± 15.77^#^
diastolic blood pressure	78.80 ± 12.41	82.04 ± 13.47	84.75 ± 11.76
Heart rate	75.40 ± 9.24	80.08 ± 10.42	76.67 ± 9.52

BMI, body mass index. ^*^P and ^**^P-value denotes significant difference between NC vs DC; ^#^P-value denotes significant difference between NC vs DEx.

### 3.2 Exercise training attenuated insulin resistance in pre-diabetic patients

Previous evidence has shown that the essence of diabetes is insulin resistance, while appropriate exercise is an effective strategy to defeat diabetes. Therefore, we first checked whether the combined Yijinjing and resistance training also have beneficial effects on pre-diabetes. The characteristics of all subjects after exercise intervention were summarized in **Table 2**. As expected, the glucose level at 2 h after glucose intake (*P*=0.007) and the value of HOMA-IR *(P*=0.020) which is generally considered to be the god standard for the assessment of insulin sensitivity, were both significantly decreased in pre-diabetic patients after exercise intervention, indicating an improvement of insulin sensitivity in response to exercise intervention in pre-diabetes. By contrast, no significant differences in fasting glucose, fasting insulin, insulin at 2 h after glucose intake and HbA1c(%) in pre-diabetic patients were found after exercise intervention. In addition, total triglyceride, cholesterol and HDL in serum samples of pre-diabetic patients all had a trend of decrease after exercise intervention without significance, probably due to that the 6-month exercise protocol is not long enough to completely restore the metabolic disorder. Taken together, the 6-month exercise intervention was able to improve insulin resistance to some extent in pre-diabetic patients.

**Table 2:** The characteristics of all subjects after exercise intervention.

Variables	NC (1)			DC (2)			DEx (3)			Group x Time		
	Baseline	Post	P	Baseline	Post	P	Baseline	Post	P	1-2	1-3	2-3
Fasting glucose (mmol/L)	5.22 ± 0.33	5.24 ± 0.29	0.334	6.30 ± 0.42	6.29 ± 0.56	0.396	6.18 ± 0.62	5.96 ± 0.41	0.255	0.591	0.813	0.121
Glucose 2h (mmol/L)	7.70 ± 2.22	6.74 ± 2.01	0.102	10.37 ± 2.16	9.30 ± 2.18	0.053	10.10 ± 2.97	8.68 ± 2.73	0.007	0.927	0.544	0.428
Fasting insulin (mmol/L)	7.45 ± 3.28	6.19 ± 2.53	0.064	11.74 ± 4.17	9.69 ± 4.09	0.119	10.89 ± 7.07	7.99 ± 3.35	0.135	0.645	0.723	0.397
Insulin 2h (mmol/L)	57.18 ± 42.31	49.42 ± 29.25	0.219	89.13 ± 58.82	84.60 ± 75.37	0.289	87.00 ± 52.15	61.89 ± 45.77	0.168	0.473	0.099	0.061
HOMA-IR	1.67 ± 0.86	1.46 ± 0.65	0.089	3.36 ± 1.31	3.14 ± 0.97	0.443	3.04 ± 2.09	1.98 ± 0.85	0.020	0.974	0.405	0.804
HbA_1c_ (%)	5.44 ± 0.33	5.44 ± 0.34	0.258	5.90 ± 0.41	5.99 ± 0.32	0.404	5.82 ± 0.45	5.97 ± 0.37	0.197	0.116	0.092	0.842
Triglyceride (mmol/L)	1.33 ± 0.72	1.03 ± 0.39	0.078	2.03 ± 1.07	1.59 ± 0.80	0.287	1.76 ± 1.07	1.70 ± 1.07	0.493	0.768	0.845	0.817
Cholesterol (mmol/L)	5.30 ± 0.94	5.30 ± 0.69	0.307	5.97 ± 1.04	5.46 ± 1.23	0.121	6.05 ± 1.18	5.69 ± 1.03	0.226	0.735	0.761	0.672
HDL (mmol/L)	1.51 ± 0.69	1.43 ± 0.23	0.217	1.44 ± 0.23	1.27 ± 0.29	0.053	1.59 ± 0.32	1.43 ± 0.30	0.064	1.30	0.178	0.146
LDL (mmol/L)	3.46 ± 0.73	3.57 ± 0.50	0.072	3.89 ± 0.78	3.74 ± 0.94	0.319	3.81 ± 0.82	3.84 ± 0.82	0.373	0.209	0.206	0.256

HOMA-IR, homeostatic model assessment of insulin resistance; HbA_1c_, glycated hemoglobin; HDL, high-density lipoprotein; LDL, low-density lipoprotein; n=16 for NC and DC group, n=21 for DEx group.

### 3.3 Exercise training alleviated liver injury in pre-diabetes subjects

**Figure 2:**
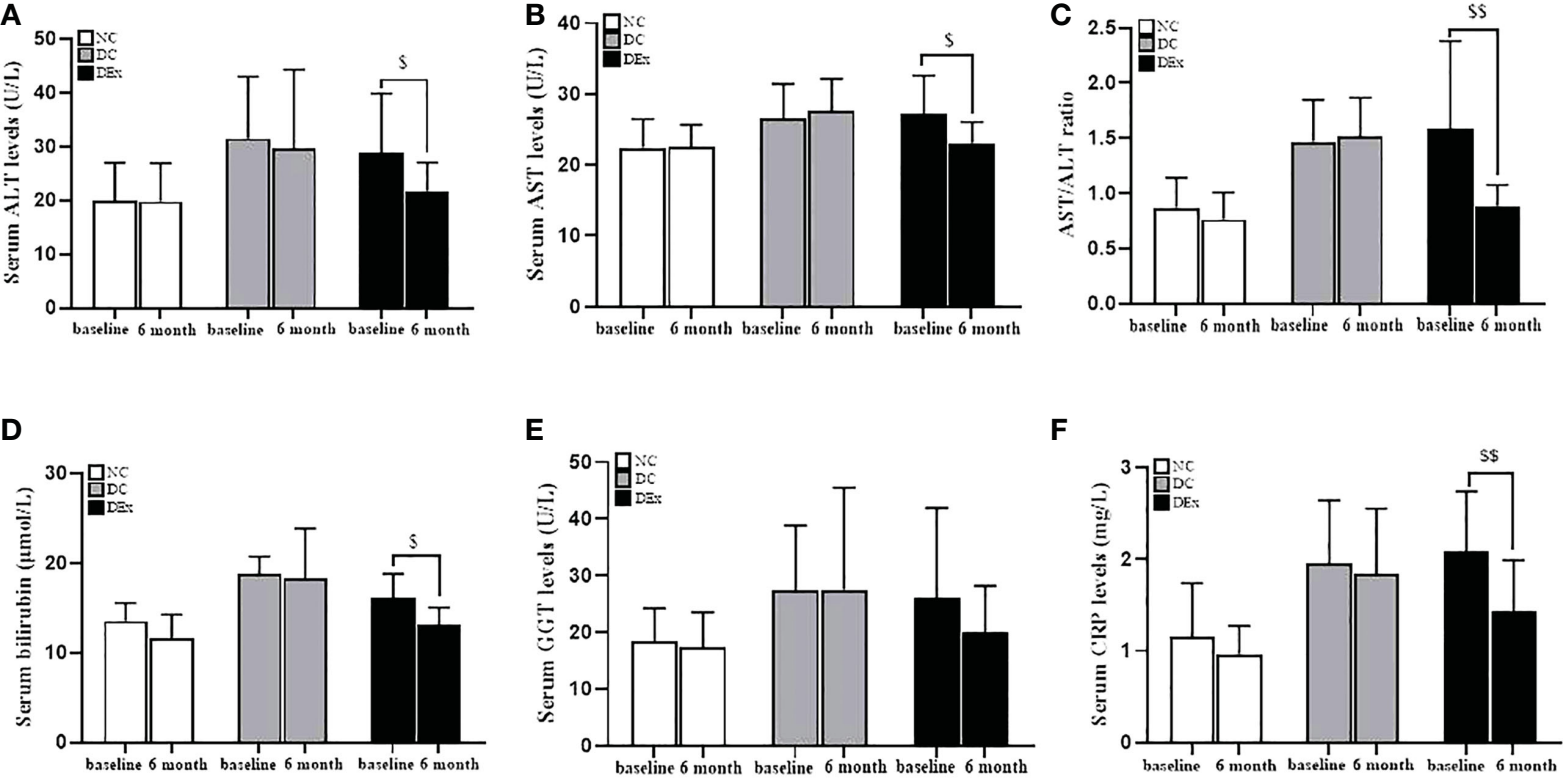
Change of liver pathology after exercise intervention. The serum levels of ALT **(A)**, AST **(B)**, AST/ALT **(C)**, bilirubin **(D)**, GGT **(E)** and CRP **(F)** were analyzed by the ELISA assay. n=16 for NC and DC group, n=21 for DEx group. $ and $$ denotes significant difference within group between baseline and after 6 month of exercise intervention. ALT, alanine aminotransferase; AST, aspartate aminotransferase; GGT, g-Glutamyltransferase; CRP, C-reactive protein.

The liver plays a central role in the systemic regulation of glucose and lipid metabolism and aberrant hepatic function is thought to be a primary driver of insulin resistance (33). As shown in **Figure 2**, the serum of ALT (**Figure 2A**), AST (**Figure 2B**), AST/ALT ratio (**Figure 2C**), bilirubin (**Figure 2D**), GGT (**Figure 2E**) and CRP (**Figure 2F**), which are the most common markers of liver injury, were all higher in pre-diabetes compared to normal control subjects. More importantly, there were significant decreases in ALT (*P*<0.05) (**Figure 2A**), AST (*P*<0.05) (**Figure 2B**), AST/ALT ratio (*P*<0.01) (**Figure 2C**), bilirubin (*P*<0.05) (**Figure 2D**) and CRP (*P*<0.01) (**Figure 2F**) after exercise intervention, suggesting that the 6-month combined exericse intervention positively affected the liver pathology in pre-diabetic patients.

### 3.4 Exercise training inhibited the overactivation of NLRP3 inflammasome in pre-diabetic patients

**Figure 3:**
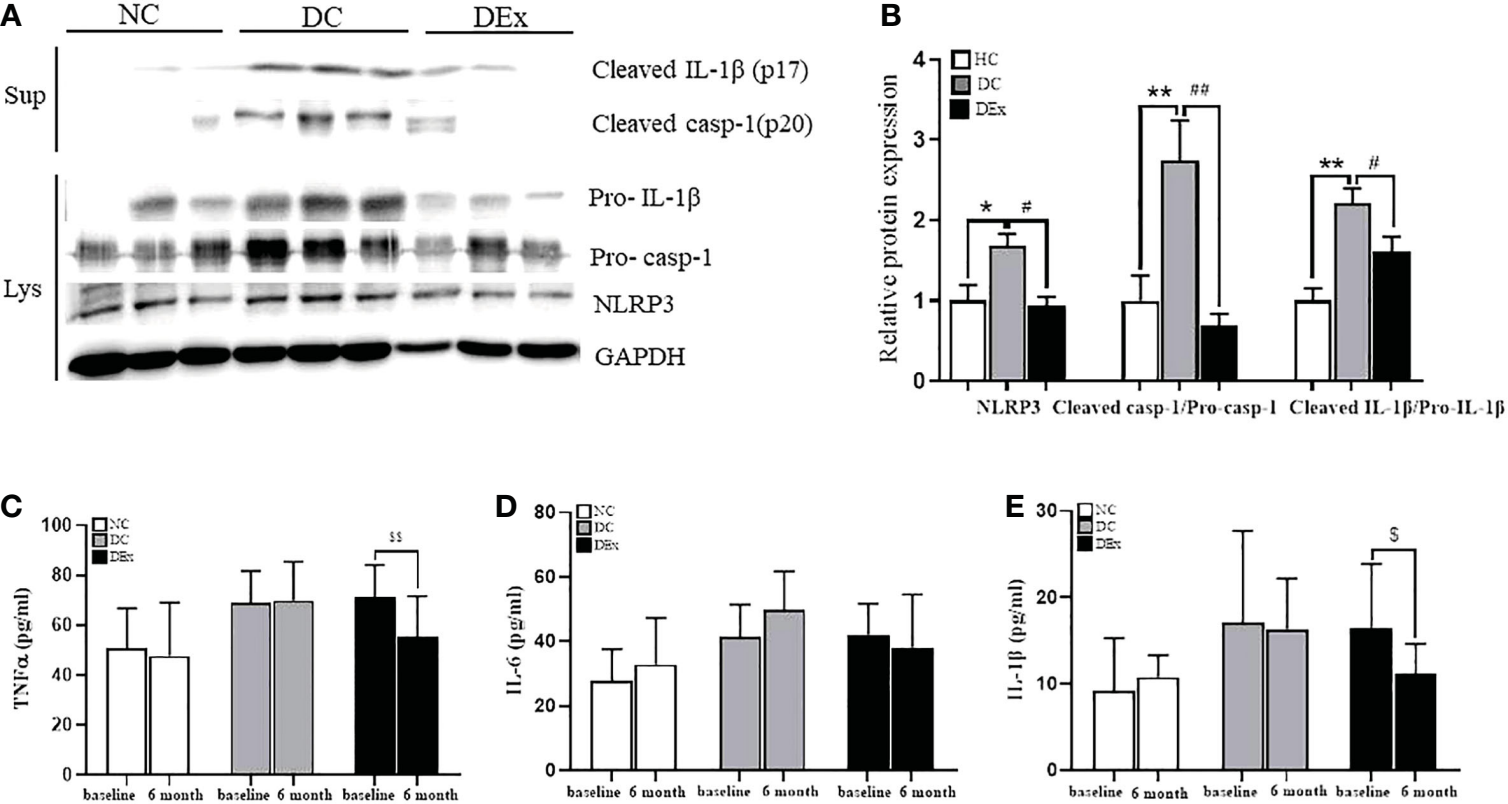
Change of NLRP3 inflammasome activity after exercise intervention. **(A)** The protein levels of NLRP3, Pro-casp-1, Pro-IL-1β in PBMCs lysate and cleaved-caspase-1, cleaved IL-1 in PBMCs supernatant of patients in NC, DC and DEx groups after 6 months of exercise intervention. **(B)** The quantification of the bands in **(A)**. **(C–E)** The concentration of TNFa, IL-6 and IL-1β in serum of patients in NC, DC and DEx groups before and after 6 months of exercise intervention. n=16 for NC and DC group, n=21 for DEx group. ^*^ and ^**^ denotes significant difference between NC and DC, ^#^ and ^##^denotes significant difference between DC and DEx. ^$^denotes significant difference between within group before and after 6 months of exercise intervention. TNFα, tumor necrosis factor; IL-6, interleukin-6; IL-1β, interleukin-1β.

As discussed above, although exercise was able to inhibit the overactivation of NLRP3 inflammasome in human models of T2DM (11), healthy young students (18), elderly subjects (19, 20), and obese children (21), few studies have focused on pre-diabetes. Therefore, we assessed the NLRP3 inflammasome activity in pre-diabetic state and verified whether exercise training could reverse these changes. As shown in **Figures 3A**, **B**, the significant overactivation of NLRP3 inflammasome in DC group was observed when compared to NC subjects, as determined by higher protein levels of NLRP3 (*P*<0.05), cleaved-caspase-1 (*P*<0.01) and cleaved IL-1β (*P*<0.01) with western blot analysis. Similarly, the serum of IL-1β, which is the end-product of NLRP3 inflammasome was also higher in DC group than NC subjects (**Figure 3E**). This finding was consistent with the elevated protein expressions of NLRP3 inflammasome signaling components. Regrettably, the prming of NLRP3 and the caspase-1 enzyme activity were not detected due to the insufficience of sample. Taken together, these results indicate that pre-diabetic patients have a low-grade chronic inflammation, which is at least partially attributed to the overactivation of NLRP3 inflammasome.

However, on the contrary, the protein expression of NLRP3 (*P*<0.01), cleaved caspase-1 (*P*<0.01) and cleaved IL-1β (*P*<0.05) (**Figures 3A, B**), as well as serum IL-1β (*P*<0.05) (**Figure 3E**) were all significantly decreased in DEx group after 6-month exercise intervention, suggesting that the 6-month exercise training is able to inhibit the overactivation of NLRP3 inflammasome, thus reducing the chronic inflammation in pre-diabetic individuals.

### 3.5 The NLRP3 inflammasome is associated with insulin resistance

**Figure 4:**
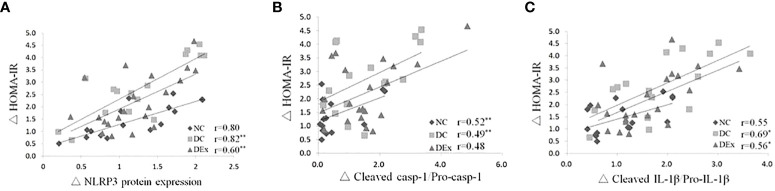
Correlations between the NLRP3 inflammasome activity and insulin resistance in the whole study population after 6-month exercise intervention. Pearson correlation tests were performed between the protein expressions of NLRP3 **(A)**, cleaved casp-1/pro-casp-1 **(B)**, cleaved IL-1β/pro-IL-1β **(C)** and HOMA-IR. n=16 for NC and DC group, n=21 for DEx group. * denotes *P*<0.05, ** denotes *P*<0.01.

Next, to determine whether NLRP3 inflammasome is involved in the regulation of pre-diabetes and exercise on systemic insulin sensitivity, we performed correlation analysis between the protein levels of NLRP3 inflammasome components and the value of HOMA-IR in the whole samples. As shown in **Figure 3**, there were positive correlations between the protein expression of NLRP3 (**Figure 4A**), cleaved casp-1/pro-casp-1(**Figure 4B**), cleaved IL-1β/pro-IL-1β (**Figure 4C**) and the value of HOMA-IR. These results suggest that the NLRP3 inflammasome activity is correlated with systemic insulin sensitivity in the context of pre-diabetes.

### 3.6 The NLRP3 inflammasome is associated with liver pathology

**Figure 5:**
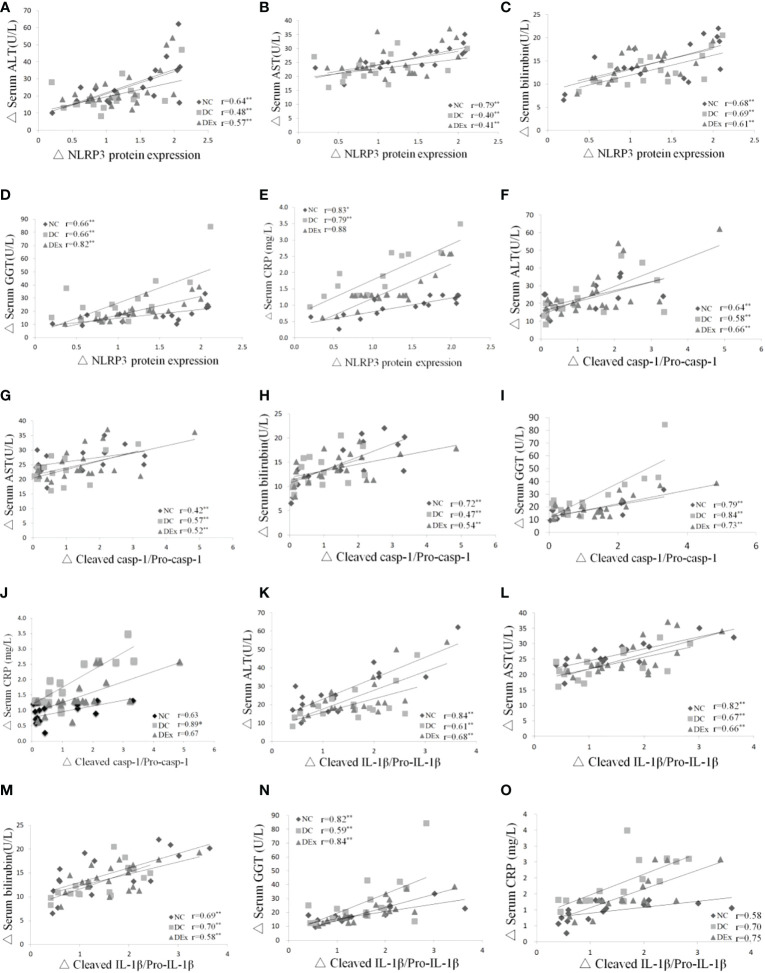
Correlations between NLRP3 inflammasome and liver pathology. Pearson correlation tests were performed between the protein expression of NLRP3 and serum ALT **(A)**, NLRP3 protein expression and serum AST **(B)**, NLRP3 protein expression and serum bilirubin **(C)**, NLRP3 protein expression and GGT **(D)**, NLRP3 protein expression and CRP **(E)**, cleaved casp-1/pro-casp-1 and serum ALT **(F)**, cleaved casp-1/pro-casp-1 and serum AST **(G)**, cleaved casp-1/pro-casp-1 and serum bilirubin **(H)**, cleaved casp-1/procasp-1 and serum GGT **(I)**, cleaved casp-1/pro-casp-1 and serum CRP **(J)**. cleaved IL-1β/pro-IL-1β and serum ALT **(K)**, cleaved IL-1β/pro-IL-1β and serum AST **(L)**, cleaved IL-1β/pro-IL-1β and serum bilirubin **(M)**, cleaved IL-1β/pro-IL-1β and serum GGT **(N)**, cleaved IL-1β/pro-IL-1β and serum CRP **(O)**. n=16 for NC and DC group, n=21 for DEx group. * denotes P<0.05, ** denotes P<0.01.

To further verify whether NLRP3 inflammasome is linked to liver pathology in pre-diabetic patients, we assessed the correlation between the protein levels of NLRP3 inflammasome components and liver injury markers ALT, AST, bilirubin, GGT and CRP in the whole samples. As shown in **Figure 5**, there were positive correlations between the protein levels of NLRP3 and serum ALT (**Figure 5A**), serum AST (**Figure 5B**) and serum bilirubin (**Figure 4C**), serum GGT (**Figure 5D**), serum CRP (**Figure 5E**). Similarly, there were positive correlations between the ration of cleaved casp-1/pro-casp-1 and serum ALT (**Figure 5F**), serum AST (**Figure 5G**) and serum bilirubin (**Figure 5H**), serum GGT (**Figure 5I**), and serum CRP (**Figure 5J**). Consistently, positive correlations were also found between the ratio of cleaved IL-1β/pro-IL-1β and serum ALT (**Figure 5K**), serum AST (**Figure 5L**), serum bilirubin (**Figure 5M**), serum GGT (**Figure 5N**) and serum CRP (**Figure 5O**). Taken together, these results suggest that the NLRP3 inflammasome is correlated with liver pathology in pre-diabetes.

## 4 Discussion

In this study, for the first time according to our knowledge, we found that 6-month combined Yijingjing and resistance training was able to alleviate systemic insulin resistance and liver injury, and inhibit the overactivation of NLRP3 inflammasome in pre-diabetic elderly subjects. More important, the NLRP3 inflammasome was positively correlated with systemic insulin sensitivity and liver injury.

Diabetes has long been associated with a chronic low-grade inflammatory state but its precise mechanisms remain unclear. In the past decade, the role of NLRP3 inflammasome in diabetes has been greatly appreciated, as the chronic low-grade inflammatory state accompanied by diabetes is at least partially consequent to the activation of NLRP3 inflammasome (34). However, whether NLRP3 could mediate the occurrence and development of diabetes by mediating the less severe pre-diabetic state is poorly understood. In this study, we found that the protein expressions of NLRP3 inflammasome components in pre-diabetic patients were higher as compared to normal healthy subjects, implying that the pre-diabetic patients were also in the chronic inflammatory state. In line with our results, one latest research also showed that the blood glucose, insulin resistance, and NLRP3 inflammasome activity were higher in pre-diabetic patients than in normal control subjects (22).

It is well established that physical exercise is an effective way to counteract diabetes but its mechanisms are not clear. Of note, in 2011, Vandanmagsar (11) first demonstrated that decreased caloric intake and exercise together resulted in reduction of NLRP3 expression in adipose tissue. Since then, studies regarding exercise intervention and the NLRP3 inflammasome are increasing rapidly. Our previous review showed that appropriate exercise exerted beneficial effects on the modulation of NLRP3 inflammasome signaling in multiple tissues of murine and human models (35). Similarly, one recent review concluded that regular exercise could significantly decrease the levels of proinflammatory factors IL-1β and interleukin-18 (IL-18), the end-products of NLRP3 inflammasome in elderly subjects (36). As mentioned above, current studies have shown that Yijinjing is effective in alleviating cognitive impairments and arthritis, however, the effects of Yijinjing on patients with metabolic diseases such as pre-diabetes are unknown. In addition, combined aerobic and resistance training is the best exercise method for elderly adults with diabetes (28). Indeed, the 8-week resistance training could prevent NLRP3 inflammasome activation and reduce apoptosis in PBMCs from elderly subjects (19). Here, we found that combined Yijinjing and resistance training for 6-months significantly reduced the protein expressions of NLRP3 inflammasome components including NLRP3, caspase-1 and IL-1β in PBMCs as well as the serum IL-1β concentration, indicating that the combined Yijinjing and resistance training were effective in relieving the robust inflammatory responses in pre-diabetes. In consistent with our findings, a 12-week strength and endurance combined training also significantly inhibited the activation of the NLRP3 signaling pathway that induced by obesity in children (21). However, another study found no significant effects of 12-month combined aerobic and resistance training on the inflammasome-related mediators in patients with combined coronary artery disease and T2DM (37). The possible reason for these contradictory findings is that the study objects in these studies are different in terms of metabolic states.

**Figure 6:**
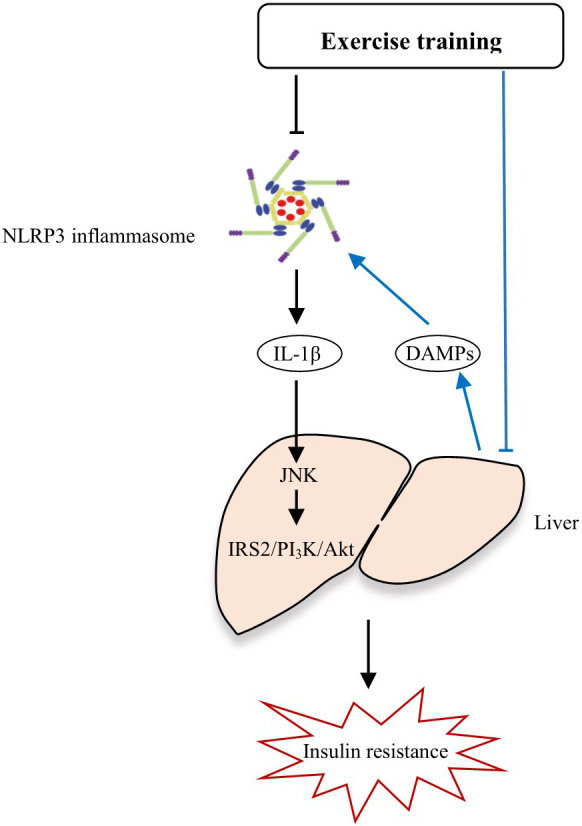
The potential mechanisms by how exercise alleviates insulin resistance and liver injury through NLRP3 inflammasome.

Additionally, we also observed significant alleviation of whole body insulin resistance and liver injury after the exercise intervention, however, whether the improvement of insulin sensitivity and liver function by combined Yijinjing and resistance training intervention is associated with the reduced expression of NLRP3 inflammasome remains unknown. Previous studies have shown that the liver is not only a central organ of energy metabolism, but also acts as an immune organ (38), as various events including metabolic stress could initiate activation of proinflammatory pathways in metabolic tissues such as liver, and then causes liver injury (39). The injured hepatocytes release DAMPs such as harmful lipids and cholesterol crystal, which then activate the NLRP3 inflammasome in hepatic macrophages and promote the production of mature IL-1β (40). IL-1β activates C-Jun N-terminal kinase (JNK), induces serine phosphorylation of insulin receptor substrate 2 (IRS-2), inhibits the expression of phosphoinositide-3-phosphate kinase (PI_3_K) and protein kinase B (Akt) protein kinase (41), thus blocking the insulin/IRS-2/PI_3_K/Akt signaling pathway in liver and leads to insulin resistance (33). In addition, the circulating PBMCs can infiltrate into the liver (42) where they become monocyte-derived macrophages, one of the major populations of hepatic macrophages (43). Therefore, the causal link between NLRP3, liver pathology and insulin resistance is complex. Exercise intervention may inhibit the overactivation of NLRP3 inflammasome, leads to a decreased level of inflammatory cytokine IL-1β, which further fails to induce hepatic insulin resistance, thus enhancing insulin sensitivity and improving insulin resistance. On the other hand, exercise may alleviate liver injury, reduces the release of harmful DAMPs from injured hepatocytes, thereby inhibiting the overactivation of NLRP3 inflammasome, which in turn, protect liver from damage (**Figure 6**). Indeed, previous study have demonstrated that the reduction of NLRP3 expression in adipose tissue was correlated with improved insulin sensitivity in obese T2DM patients, and they further confirmed that ablation of NLRP3 in mice prevented the obesity-induced inflammasome activation in fat depots and liver together with enhanced insulin signaling, suggesting that exercise may improve insulin sensitivity by inhibiting NLRP3 inflammasome activation, thus alleviating diabetes (11). Similarly, a positive correlation between insulin, HOMA-IR and IL-18 expression in adipose tissue and an inverse correlation between glucometabolic variables and leukocyte expression of NLRP3 and caspase-1 were observed in elderly subjects (19). Consistent with these findings, our study found tight correlations between the protein expressions of NLRP3 inflammasome components and insulin resistance, and liver pathology in elderly pre-diabetic subjects. However, a recent study found that insulin resistance and liver histopathology in metabolically unhealthy subjects did not correlate with the hepatic abundance of NLRP3 inflammasome nor circulating IL-1β levels (44). The most likely reason for the discrepancy may that the objects in this study were less obese individuals.

To sum up, we speculated that the combined Yijinjing and resistance training may be an effective strategy to inhibit the robust NLRP3 inflammasome activation, thereby alleviating liver injury and insulin resistance in pre-diabetes. Apart from this mechanism, the combined Yijinjing and resistance training may also alleviate liver injury, reduce the release of harmful DAMPs from injured hepatocytes, thereby inhibiting the overactivation of NLRP3 inflammasome (**Figure 6**). However, there are some issues remains unanswered. Current studies show that the underlying mechanisms responsible for the effects of exercise on insulin resistance and fatty liver are complex and elusive. Exercise exerts its effects on insulin resistance and liver metabolism at multiple levels. Specially, regular exercise increases the insensitivity of insulin to increase glucose transport into muscle, inhibit lipolysis in adipose, and inhibit hepatic glucose output (45). Of note, studies in the past decade demonstrate that the aberrant activation of NLRP3 inflammaosme is a key driver for diabetes, while regular exercise could suppress the excessive NLRP3 inflammasome activation, which may be a mechanism underlying the beneficial effects elicited by exercise in diabetes (35). However, very regrettably, there are few studies about the effects of exercise on NLRP3 inflammasome so far and the mechanisms by which exercise regulates NLRP3 inflammasome activation are largely unknown. Given that various endogenous signalings including mitochondria, lysosomal disruption, Golgi disassembly, cellular ion flux and metabolic changes are involved in NLRP3 inflammasome activation (10), it is easy to speculate that exercise may mediate NLRP3 inflammaosme activity through affecting these endogenous signalings, but few studies have ever explored this hypothesis, and more evidences are needed to address this issue.

Apart from liver, the skeletal muscle is another major metabolic organ. Moreover, increasing evidence suggests that inflammation occurs in skeletal muscle is mainly manifested by increased immune cell infiltration and proinflammatory activation. By secreting proinflammatory molecules, the immune cells may induce inflammation and insulin resistance in skeletal muscle (46). Indeed, since the cytokine IL-6 was first found to be secreted into the bloodstream in response to muscle contraction in 1998 (47), various cytokines secreted by skeletal muscle have been identified (48). Among all the identified cytokines, apelin, BDNF, CTSB, CXCL1, FGF21, Follistatin, GDF-15, IL-6, IL-8, IL-10, irisin, lactate, METRNL, reactive oxygen species have been shown to be related with the NLRP3 inflammasome in various models. Taken together, the skeletal muscle is an endocrine organ and secretes various cytokines, which then probably activates NLRP3 inflammaosme, thus promoting the chronic low-grade inflammation state in skeletal muscle at least to an extent. But whether exercise could improve systemic metabolism and inhibit NLRP3 inflammasome activation through affecting the cytokine secretion from skeletal muscle is unclear, future studies should consider the potential role of muscle during exercise affecting systemic metabolism and NLRP3 inflammasome activity.

Strengths of our study include the objects of study, intervention protocol. The objects of our study are pre-diabetic populations. Although it is wise to ameliorate diabetes by developing an effective strategy for the pre-diabetes, no RCTs are available to evaluate the effects of Yijinjing in patients with metabolic diseases such as pre-diabetes. Since data from long-term studies that show the effects of combined Yijinjing and resistance training on pre-diabetic are currently lacking, the findings from our study may have significant clinical implications for diabetes because they show that if healthy lifestyle intervention is established at the early stage of diabetes they can prevent diabetes progression.

Our studies have some limitations. First, the sample size of this study is relatively small and thus may not be fully representative of the general pre-diabetic elderly population, therefore, randomized controlled studies with larger sample sizes are needed to confirm these findings in the future. In order to obtain accurate information regarding the degree of inflammation and liver pathology, liver biopsies would be needed. However, given the ethical issues, it is hard to obtain biopsies from patients, thus limiting the direct measurement. Additionally, in this manuscript, we combined the data from males and females. For precision medicine, it would be meaningful to study the function of exercise training in males and females separately. Lastly, although we found positive correlations between insulin resistance, liver pathology and NLRP3 inflammasome, however, it should be noted that correlation does not imply causation. Therefore, more research including mouse model is needed to define the role of NLRP3 inflammasome in pre-diabetic populations in the future.

## 5 Conclusion

Taken together, our findings indicate that combined Yijinjing and resistance training is effective in alleviating insulin resistance and liver injury in elderly pre-diabetes, which is associated with the inhibition of NLRP3 inflammasome activity. The findings of this study have significant clinical implications for pre-diabetic patient since early prediction of diabetes is vital for timely intervention.

## Data availability statement

The original contributions presented in the study are included in the article/supplementary material. Further inquiries can be directed to the corresponding authors.

## Ethics statement

The studies involving human participants were reviewed and approved by Ethics Committee of Shanghai University of Sport. The patients/participants provided their written informed consent to participate in this study.

## Author contributions

TZ designed the experiment, participated in data collection and analysis and drafted the manuscript. JT was responsible for exercise intervention and participated in data collection. JF participated in data collection. XL and RW designed the experiment and oversaw the implementation of the project. All authors contributed to the article and approved the submission.
